# The Week's Work

**Published:** 1911-01-28

**Authors:** 


### The Week's Work.

### HOSPITAL GUIDE-BOOKS.

An item in the half-yearly accounts of the Ipswich and East Suffolk Hospital was £56 16s. for an illustrated Guide Book. This sum was apologised for by Mr. F. Messent, who presented the financial statement, on the ground that the advertisements which it contained brought in a substantial sum. We hope that no one who attended the half-yearly meeting required any apology for the cost of producing such a book. Hospital Guides, whether accompanied by many advertisements or not, are such an invaluable method of attracting public interest to hospitals that they may be regarded more justly as a sound investment than as an item of expense. The fact is that even to-day the public at large remains ignorant of the appearance of a modern hospital ward or theatre, and the only way of overcoming this is to issue such a Guide as that to the Ipswich Hospital, which may be regarded as a capital example. The inside of a modern hospital is cheerful enough, but the outside is, we are afraid we must admit, often dour and depressing. Till the day comes when hospital architects are able to meet reasonable æsthetic demands on the exterior of their buildings, illustrated hospital Guides will remain the best way of overcoming ignorant prejudice against them, and of attracting public sympathy and interest.

### A RECORD OF PROGRESS AT BRADFORD.

The files of The Hospital contain a detailed record of hospital progress, and the pursuit of references to one institution in the index reflects, sometimes in an amusing way, the story of institutional development. The latest example of this weekly record concerns the Bradford Royal Infirmary, and we are able this week to congratulate the authorities of this institution on having so far proceeded with their statesmanlike plan of reconstruction as to invite designs for the new building which the City of Bradford most certainly deserves. The bare fact is given in our new section "Works Contemplated and in Progress," but that fact needs a reference to earlier issues if its importance is to be understood. More than a year ago—to be precise, on November 6, 1909 (page 161)—we pointed out that "for fifteen years the Bradford Royal Infirmary has urgently required to be rebuilt," and we were then happy to record the public spirit of the Lord Mayor, which had been the mainspring of the successful appeal fund, that reached already £40,000 at the time of writing. On February 12 of last year, page 584, the decision of the infirmary authorities to purchase the Daisy Hill site is noted. Now we are able to say that Duckworth Lane is to be built on for the purposes of extending the institution. The fact that the great work of reconstruction, so badly needed, has shown this steady progress in the face of many difficulties is one more sign of the high standard which the cities of the North are demanding with such consistency in their great hospitals. As much as any they have learnt to follow the late King's example in this respect, for in the proposed County Hospital Fund which was launched successfully at Leeds last week, the care which was taken by its promoters not to injure existing funds should be a lesson to certain institutions which have hampered appeals in the past by the want of this institutional solidarity. The Bradford Royal Infirmary had this as one of its great difficulties a year ago, and we can only repeat now what we said then, that the amalgamation of institutions which time has made merely competitive is the statesmanlike solution of such struggles. We believe that the rebuilding of the Bradford Royal Infirmary will probably mark an epoch in hospital construction, and may even be the first step towards the inevitable goal of hospital development, which is the hospital city.

### OUT-PATIENT CLASSIFICATION.

The satisfactory classification and indexing of out-patients are often matters of great importance, and, owing to the difficulty of satisfactorily dealing with them, as often a source of worry to hospital officials. The number of methods recommended shows that no one system can claim to be perfect, and every day almost one hears of a new method or a new means of applying old principles. We have just had the opportunity of hearing about the excellent way in which such classification is accomplished at the Leeds General Infirmary. The card method is used there, the cards being filed in a cabinet in labelled drawers, which makes it comparatively easy to find a name commencing with a letter of the alphabet corresponding to that on the label. The card of admission gives the following particulars: Name, address, employment, name of employers, father or husband, medical officer, age, married or single, rent, wages, family income, sick club, number of family, and date. The final out-patient index card gives further details regarding the treatment, its duration and completion, whether patient was cured, sent to the workhouse infirmary, asylum, or fever hospital, and particulars about the nature of the complaint. These out-patient cards, it seems to us, should prove of great value for statistical purposes in a couple of years. They give accurate and more or less adequate particulars on just the points on which the sociologist desires to be informed, and we imagine that a study of the records at the out-patient department of the Leeds Infirmary ought to yield interesting data. The system is certainly deserving of consideration by the authorities of other hospitals.

### THIS WEEK'S DRUG MARKET.

Business in the drug market continues fairly good; there have been few actual changes in prices, although in the case of a number of drugs the tendency of values is in an upward direction. As was not altogether unexpected, the price of Japanese refined camphor is advancing, but at present there is no change in English refined. Menthol is again dearer, and peppermint oil is likely to advance.

There seems to be no likelihood of any diminution in the value of olive oil, which is fetching extreme prices; reports from the producing districts are of an unfavourable character, and the period of scarcity is likely to be prolonged. Ergot of rye continues to rise in price, and present quotations are more than twice as high as those ruling a few months ago. The high price of oil of cubebs has had the effect of encouraging the practice of adulteration, and buyers should therefore be cautious. Cod fishing has commenced in Norway, but up to the present there is little to indicate whether the season is likely to be a good one or otherwise.

